# Analysing public goods games using reinforcement learning: effect of increasing group size on cooperation

**DOI:** 10.1098/rsos.241195

**Published:** 2024-12-11

**Authors:** Kazuhiro Tamura, Satoru Morita

**Affiliations:** ^1^ Department of Environment and Energy Systems, Graduate School of Science and Technology, Shizuoka University, Hamamatsu 432-8561, Japan; ^2^ Department of Mathematical and Systems Engineering, Shizuoka University, Hamamatsu 432-8561, Japan

**Keywords:** public goods game, reinforcement learning, Q-learning, agent-based model

## Abstract

Electricity competition, restrictions on carbon dioxide (CO2) emissions and arm races between nations are examples of social dilemmas within human society. In the presence of social dilemmas, rational choice in game theory leads to the avoidance of cooperative behaviour owing to its cost. However, in experiments using public goods games that simulate social dilemmas, humans have often exhibited cooperative behaviour that deviates from individual rationality. Despite extensive research, the alignment between human cooperative behaviour and game theory predictions remains inconsistent. This study proposes an alternative approach to solve this problem. We used Q-learning, a form of artificial intelligence that mimics decision-making processes of humans who do not possess the rationality assumed in game theory. This study explores the potential for cooperation by varying the number of participants in public goods games using deep Q-learning. The simulations demonstrate that agents with Q-learning can acquire cooperative behaviour similar to that of humans. Moreover, we found that cooperation is more likely to occur as the group size increases. These results support and reinforce existing experiments involving humans. In addition, they have potential applications for creating cooperation without sanctions.

## 1. Introduction

Even when non-cooperation is the optimal behaviour in game theory, some humans behave cooperatively when playing public goods games [1–6]. Numerous studies have explored explanations of players’ cooperative behaviour within the framework of game theory and conducted experiments that are applied in other areas, such as psychology, management, economics and organizational theory [3–9]. Notably, recent studies reported that cooperation is promoted when the group size is large, although game theory has shown that non-cooperation is a rational behaviour regardless of the group size in a public goods game [10–17]. There remains no explanation for how larger group size promotes cooperative behaviour, owing to the limitation of group size to dozens at most human experiments. Hence, this study assesses the behavioural mechanisms that cause people to cooperate when the group size increases in public goods games.

It is challenging to increase the group size when conducting human experiments that address this issue. The larger the scale of the public goods game and the more realistic its implementation, the greater the need to address costs, time constraints, cultural backgrounds, regional characteristics and ethical considerations associated with the experiment. Thus, reproducibility is an issue when explaining the mechanisms of group size and cooperative behaviour through experiments with humans. The results of the public goods game on a large scale have been reported [16]; however, Balliet *et al*. [18] stated that in 76 papers on public goods game and prisoner’s dilemma experiments, the average group solely comprised approximately four. This fact indicates how the cost of experimentation poses a significant obstacle in investigating public goods gaming on a large scale. In addition, humans have individual differences and the reasons for their actions are too complex, making it difficult to analyse their thoughts. Thus, even if one derives a hypothesis for the mechanism, it is challenging to verify whether it is generally appropriate. To address these problems, there is a need for a simpler and easier model to analyse than humans, which is a simulation method that can repeatedly verify large-scale public goods games.

In this study, we used a well-established method, deep Q-learning (DQL) [19]. DQL is a type of reinforcement learning that comprises learning the optimal strategy for a situation from experience and has been extensively used in the past decade. DQL is an extension of the Q-learning method involving the learning of the expected gain (Q-value) in the future when ‘a certain action’ is taken in ‘a certain situation’ using a deep neural network (DNN; [19–22]). Reinforcement learning may produce better results than traditional deterministic methods in behavioural psychology and robotics which include strategic behaviour and, in some cases, achieve greater capabilities than humans [23,24]. Despite reinforcement learning being a fundamental technology in artificial intelligence (AI), recent focus has shifted towards cooperation research using natural language processing models, surpassing traditional reinforcement learning methods in popularity [25–28]. This study selected DQL, which is a reinforcement learning method, for three reasons. First, it can clarify the mechanisms that cause cooperation in large-scale public goods games, because the agents’ behaviour is determined by the Q-value, rather than by a complete black box. Second, Q-learning has proved beneficial in existing research [29,30]. Finally, the concept of the Q-value is similar to that of the sum of the discounted expected gains in conventional iterative games [31]. The difference between the two is whether the sum of the discounted expected gains is calculated according to the behaviour of rational players or by an experience-based Markov process.

This study aimed to clarify how cooperative behaviour mechanisms are acquired. If the DQL method allows the agent to acquire cooperative behaviour, the result would overturn the idea that non-cooperation is rational, as indicated by existing game theory, and suggest that both humans and AI can acquire cooperative behaviour from experience. Our experimental results indicate that cooperative behaviour remains evident even after a sustained period of learning. A correlation between group size and cooperative behaviour was observed, similar to the behavioural experiments [10–17]. We conclude that as the group size increases, the Q-values for choosing cooperative and non-cooperative actions become relatively close, thereby increasing the likelihood that cooperation will be maintained. Thus, we have identified a rationale for the relationship between cooperative behaviour and group size that could not be explained by within the framework of traditional game theory.

## 2. Material and methods

DQL is a type of Q-learning in which Q-values are calculated using a finite table called a Q-table, which is calculated using a neural network. DQL is suitable for a large group size because it can continuously learn Q-values, which Q-tables can only learn in discrete situations. Furthermore, it can compute high-dimensional problems [19]. Thus, in this study, experiments were conducted by replacing humans with a reinforcement learning agent using DQL (DQL-agent).

This study considers the N-person public goods game with two strategies. Public goods games were simulated using agents trained in reinforcement learning with DQL, rather than human participants. Each DQL-agent chooses whether to invest c=1, and the total is multiplied by b before being distributed equally amongst the N player parts. The actions that pay the cost are denoted as cooperation (C), while the actions that do not pay the cost are denoted as defection (D). For simplicity, DQL-agents decide their actions using the cooperation percentage of the other agents in the previous round. This approach is rooted in the well-known effectiveness of the tit-for-tat strategy, which has only one memory [32]. In most experimental situations, participants knew and responded to the cooperation percentage based on the gains they received. The two Q-values ($Q_{C}$ and $Q_{D}$) are functions of the previous action of the focal agent and the cooperation percentage of the other agents in the previous round. The agent then takes an action with the higher Q-value.

The Q-value is the expected gain predicted by three factors: the previous action of the focal agent, the cooperator’s percentage around it and the action it is about to take. According to Watkins & Dayan [20], the Q-value was calculated using the following equation:


(2.1) $$Q^{\pi }\left(s_{t-1},a_{t}\right)=\;\sum_{s_{t}\in S}P\left(s_{t}|s_{t-1},a_{t}\right)\left\{r_{t}+\gamma max\left(Q^{\pi }\left(s_{t},C\right),\right)Q^{\pi }\left(s_{t},D\right)\right\},$$

where $Q^{\pi }(s_{t-1},a_{t})$ represents the Q-value when taking action $a_{t}$ in the situation $s_{t-1}$. The action is $a_{t}=C$ or D and the situation is represented as $s_{t-1}=\{a_{t-1},p_{t-1}\}$, where $p_{t-1}$ stands for the cooperator percentage around the focal agent. $P(s_{t}|s_{t-1},a_{t})$ represents the probability of being $s_{t}$ if $a_{t}$ is taken in the situation $s_{t-1}$, which varies with the opponent and expresses the opponent’s behavioural philosophy. The discount factor (0≤γ≤1) parameters the importance of the expected future Q-value relative to the immediate gain $r_{t}$. γ is also the probability that the game will be repeated the next period. γ=0.9 means that there is a 90% probability that the next period will also take place. Hence, on average, the public goods game will be repeated for 10 periods. Small γ-values make agents more ‘short-sighted’ and focused on immediate rewards $r_{t}$. In some prior studies using DQL, γ is close to 1, such as γ=0.99 [19], but most studies use iterated public goods games with 10−20 steps; γ is set to 0.9, unless otherwise noted. The immediate gain $r_{t}$ represents the income obtained as a result of a certain action in a certain situation and is defined for focal agent i as follows:


(2.2) $$r_{t}=\;-g_{i}+\frac{b}{N}\sum_{j=1}^{N}g_{j},$$

where $g_{i}=1$ and $g_{i}=0$ if agent i cooperates and defects, respectively. Equation (2.2) is a common gain formula used in general public goods games. Here, we focus on the magnitude of return on an individual’s investment:


(2.3) $$\alpha =\frac{b}{N},$$

which is called marginal per capita return (MPCR). In general, experiments with humans are conducted by first defining α and the group size N of the public goods game. We consider the range 1/N<α<1 where the dilemma exists.

We now explain the composition of the deep Q-network (DQN), which is the DNN that determines the behaviour of the DQL-agent. The role of the DQN is to approximate the calculation of the Q-values through learning. First, the DQN receives $s_{t-1}=\{a_{t-1},p_{t-1}\}$ as input. Subsequently, we obtain $Q_{C}=Q^{\pi }(s_{t-1},C)$ or $Q_{D}=Q^{\pi }(s_{t-1},D)$ as the output. The DQL-agent subsequently compares $Q_{C}$ and $Q_{D}$ calculated by the DQN and selects the larger of the two as the next action. To obtain an approximate calculation of the Q-values, the DQL-agent learns by repeating 30 games of 20 periods per game, during which the ε-greedy method is used to mix random actions in each period of the game to search for Q-values, and from the middle of the game onwards only the almost optimal behaviour is taken [29,30]. The specific ε is expressed by the following equation using the number of game iterations t. This probability is used to select a random move instead of an action based on the Q-value.


(2.4) $$\varepsilon =0.001+\frac{0.25}{1+t}.$$

**Figure 1.**
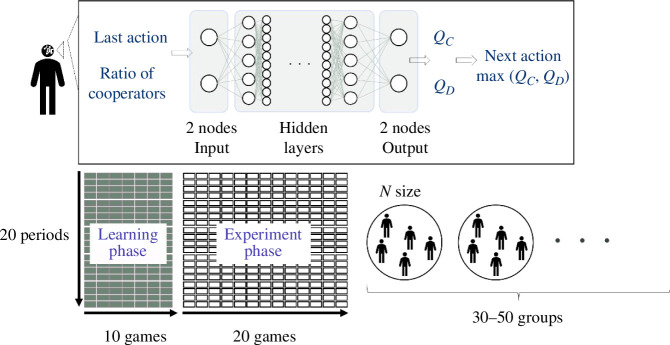
Design of public goods game and DQL-agent. The design of the public goods game and the neural network structure of the DQL-agent are shown here. The public goods game consists of 20 periods and is repeated 30 times. The first 10 games were used for training and the last 20 games were used for the experimental results. A group consisted of N DQL-agents and the average cooperation rate was investigated by preparing 30−50 groups and playing the game in parallel.

The experiments in this study were conducted by defining the group size N and MPCR α. We created N DQL-agents and organized them into a group. Public goods games were conducted over 20 consecutive periods per game, with each DQL-agent playing 30 games. However, the initial 10 games, comprising 200 periods, were excluded from the analysis as a learning phase. In each period, all DQL-agents in the group determine their behaviour based on the situation $s_{t-1}$ and receive the immediate gain defined by equation (2.2). The DQL-agents then calculate and memorize the Q-value using equation (2.1). Through this process, they learn the best action in a given situation in every period. The experiment was conducted with 30−50 groups, except for extreme cases such as *N* = 1000 (figure 1). The parameters described in §2 are summarized in table 1. The parameters of the DQN are optimized by Optuna based on pre-training data when each agent makes a random move [33].

**Table 1.** Parameters used in the experiment.

parameter	description
α	MPCR, 0≤α≤1
γ	the discount factor (0.6≤γ≤0.99); the importance of the expected future Q-value for the immediate gain
N	the number of DQL-agents in a group or the size of group, 2≤N≤1000

## 3. Results

**Figure 2.**
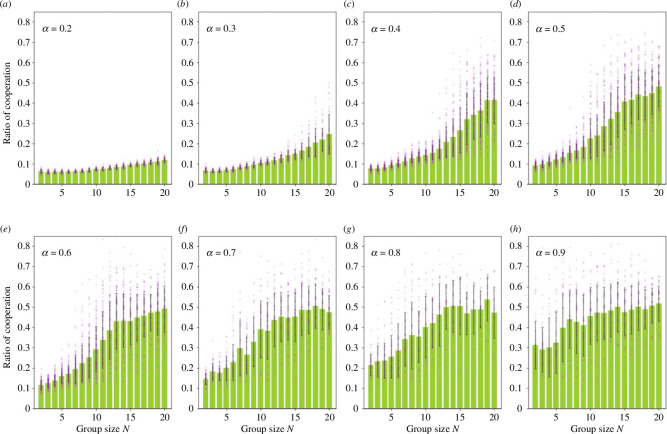
Relationship between the group size and percentage of cooperation. The vertical axis is the fraction of DQL agents choosing C, and the horizontal axis is the group size N. Moreover, (*a*) represents results for marginal per capita return α=0.2, (*b*) for α=0.3, (*c*) for α=0.4, (*d*) for α=0.5, (*e*) for α=0.6, (*f*) for α=0.7, (*g*) for α=0.8 and (*h*) for α=0.9. The purple dots represent the percentage of cooperation for each group. The green bars represent the average of cooperation, and the error bars represent the standard deviation of the 50 groups.

Figure 2 shows the change in the percentage of cooperators when the number N of the DQL-agent is changed, wherein the percentage of cooperators tends to increase as N increases. Additionally, the increase in the cooperation rate does not increase suddenly after a certain threshold but rather continuously as N increases. If the cooperation rate is less than 50%, it increases as the group size increases; however, as it approaches approximately 50%, the growth of the cooperation rate slows down. Specifically, in the range of 0.4 ≤ α ≤ 0.7, the cooperation rate reaches 45% at 12 ≤ N ≤ 20 and the growth slows down. Therefore, the boundaries that can promote cooperation exist at the above α and N. In addition, when the average cooperation rate is small, there is no group with an outstandingly high cooperation rate and the variance is small. However, when the average cooperation rate exceeds 20−30%, a group in which approximately 60% or more of the DQL-agents choose to cooperate emerges and the variance is large.

**Figure 3.**
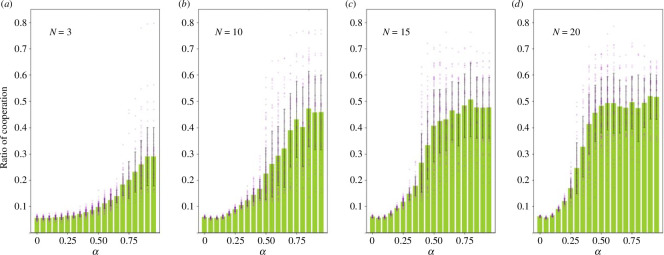
Relationship between marginal per capita return (MPCR) and cooperation. As shown in figure 2, the vertical axis represents the percentage of cooperators, and the horizontal axis represents the MPCR: 𝛼. The purple dots represent the percentage of cooperation for each group, while the green bars represent the average of cooperation. The error bars represent the s.d. of the 50 groups. Moreover, (*a*) shows the results for 𝑁 = 3, (*b*) for 𝑁 = 10, (*c*) for 𝑁 = 15 and (*d*) for 𝑁 = 20. Almost all results illustrate that an increase in the MPCR promotes cooperation.

Subsequently, we show the results when α increases. Figure 3 indicates that the percentage of cooperators increases with α even if α<1. As mentioned earlier, when α<1, defection is more profitable than cooperation regardless of the surrounding cooperation rate. Figure 3 also shows that, as in figure 2, the cooperation rate stops growing when it reaches approximately 45−50%, and thereafter, it is not affected much by the increase in α.

**Figure 4.**
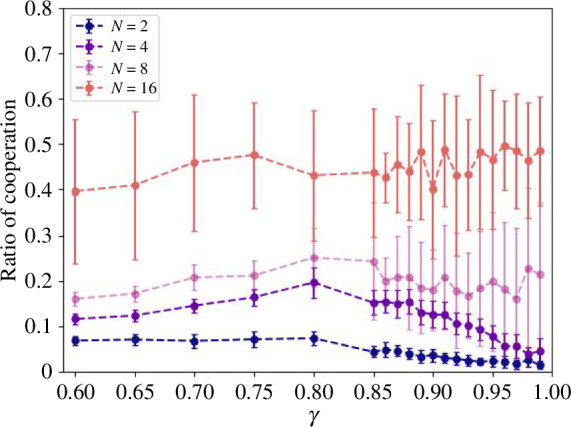
Cooperation rate for varying the discount factor γ. It shows the change in the cooperation rate when the discount factor γ is varied. The vertical axis is the cooperation rate, and the horizontal axis is the discount factor γ. Broken lines are for eyes and each line represents the results for N=2, 4, 8 and 16. The error bars represent the s.d. It can be seen that for any γ, the cooperation rate is larger for larger N.

The above results show that the promotion of cooperation by changing the group sizes N and α may be from the use of Q-values, which may depend on the discount factor γ used to calculate Q-values. Figure 4 shows the results when γ is varied to confirm the robustness of the results. Figure 4 describes the result that the cooperation rate increases as N increases for all values of γ, confirming a robust result.

**Figure 5.**
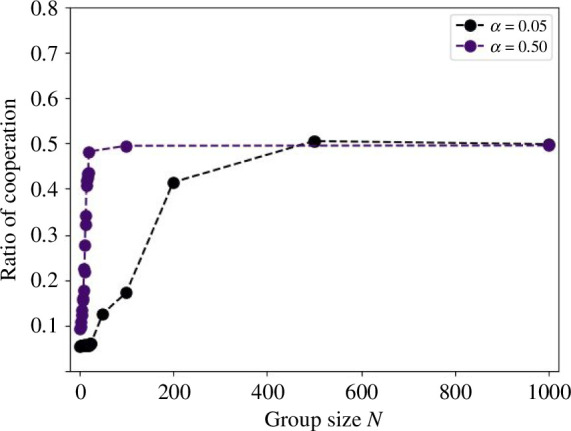
Cooperation rate when the number of DQL-agents is large. This figure shows the change in the cooperation rate when the DQL-agent is increased up to N=1000. The vertical axis represents the cooperation rate and the horizontal axis represents the group size N.

This raises questions regarding the results if N were made extremely large. Because it was difficult to experiment with 50 groups in parallel for the public goods game with a large N owing to the execution time, the number of groups was limited, as presented in table 2. Figure 5 shows the experimental results for α=0.05 and α=0.5, increasing the size up to N=1000. No substantial difference existed between α=0.5, N=100 and α=0.05, N=1000, where b=50. These results suggest that the upper limit of the cooperation rate was approximately 50%.

**Table 2.** Number of groups used in the experiment.

N	2−20	21−25	50	100	200, 500	1000
α=0.05	50	0	30	30	10	10
α=0.50	50	30	0	10	0	10

## 4. Discussion

This study investigated the effects of group size on cooperative behaviour using DQL-agents. We obtained three key findings. First, we discovered that cooperative behaviour could persist when using the Q-learning method. Second, we observed that as group size increases, the rate of cooperation also increases, consistent with studies conducted with humans [10–17]. Finally, we found that once the group size exceeds a certain threshold, the increase in the cooperation rate stagnates approximately 50%. Subsequently, we discuss why the cooperation rate approaches 50% as the group size N increases. The computational errors by the DQN cause cooperation to survive. This is because public goods games become lower risk and higher return as the number of players increases. Thus, the difference between the maximum and minimum pay-offs for this game is α(N−1), which is substantial when N is large. However, the gain by choosing defection in each round is only (1−α) larger than the gain by choosing cooperation. Therefore, the difference in pay-offs between choosing C and D may be negligible and the DQL-agent may cooperate with a probability of 50%. Assuming a scenario where opposing agents randomly choose between two strategies, the Q-value can be analytically derived from equation (2.1). We can also evaluate the difficulty of learning as $\left(1-\frac{Q_{C}}{Q_{D}}\right)$. For example, when N=2 and α=0.2, we can calculate the difficulty as 0.8, indicating that the *D* strategy is superior as this value is close to 1. However, in threshold cases such as N=20 and α=0.5, or N=8 and α=0.9, the difficulty is approximately 0.01, suggesting minimal difference between $Q_{C}$ and $Q_{D}$. As shown in figure 2, DQL-agents make random choices in such cases. The threshold value of the difficulty depends on the learning time or the number of layers in the DNN. Nonetheless, achieving zero error using DQL-agent is not feasible. Thus, we found that as the group size or MPCR increases, the difference in Q-values between cooperative and non-cooperative diminishes, leading agents to perceive similar gains regardless of their choice. Additionally, as shown in figures 2 and 5, the relationship between group size and cooperation rate is not linear, suggesting that a group size of a dozen or so is optimal in cases where the larger the group size, the greater the cost of maintaining the society.

We compare this study with existing studies involving humans. The results in figure 2 are consistent with those reported in previous studies [14,15]. They reported lower cooperation rates with N=4 compared with N=40. The difference in the setting is that the previous studies allowed participants to select any amount for their contribution, whereas our study restricts agents to choosing between two options. In the previous study, the average investment exceeded half of the available amount at N=40 [14]. Although this study revealed that the cooperation rate is approximately 50% owing to learning by experience, humans may learn to cooperate more and resolve dilemmas owing to social norms and other factors. However, the effects of social norms and other factors are unclear when group size is further increased. Pereda *et al*. reported a negligible difference in the amount invested when N=100 and N=1000 [16], aligning with this study’s results in figure 5. However, existing studies have different arguments for the results, as shown in figure 5, such as that the cooperation rate converges at 50% even when N=1000. Capraro & Barcelo reported a decrease in cooperation rate after a certain size [15]. Nonetheless, their experiment differed from our setting, in which the gain in the public goods game kept increasing with the number of cooperators, whereas the gain was constant when there were more than a certain number of cooperators. Therefore, the results may differ depending on the experimental setting. Our results support the assertion of Pereda *et al*.; however further investigation is needed to determine the results in a setting similar to that of Capraro & Barcelo. Compared with other studies, similar results have been reported by Zelmer in a meta-analysis of experiments with humans, where cooperation was promoted as α increased [34]. Isaac *et al*. also reported a positive effect on group size and increased average cooperation when α was 0.3, but not when MPCR was 0.75; the results are similar for the convergence of cooperation rates at smaller N as α increases [12]. However, the cooperation rate did not reach 50% for small group sizes, such as three, which quantitatively highlights the limitations of the model in this study. Overall, the model with DQL partially replicates the experiment with humans.

Finally, this study identifies several future research challenges. First, while it is widely accepted that an effective public goods improvement model should achieve multiple steady states through the adjustment of system parameters, our model struggled to identify these states. Given that our model was developed to replicate human experiments, we predict that the necessary information for learning the steady state was not input into the DQL-agent. For example, although participants are not explicitly informed about the period they are in during the human experiment, this information is implicitly relevant to their decision-making and is crucial for the formation of the steady state. We demonstrated that the evolution of cooperation, using a simpler model, results from erroneous learning owing to the small difference between $Q_{C}$ and $Q_{D}$. To clarify the relationship between group size and cooperation rate, we will develop a more complex model that achieves the steady states. Second analytically calculating Q-values when multiple DQL-agents are used presents a significant challenge. Currently, we rely on approximations using neural networks owing to the complexity of the Q-value calculations. However, if we achieve analytical solutions, it might lead to a more rigorous description of the relationship between the group size and cooperation rate. Consequently, this can clarify the relationship between our study and conventional evolutionary game theory. The third challenge is to make our study useable for more complex realistic situations. Here, we simplified the input values used to calculate Q-values so that they can be partially compared with the analytical values. However, we aimed to create a more realistic agent by using more input values. If our results obtained in a simple public goods game hold in real situations, there may be an appropriate number of people in a group with whom cooperation is successful. It is common for groups of approximately 20 people to work on a task. For example, an elementary school class contains 20−30 students, an army platoon comprises 20−50 soldiers and a workplace section contains 15−20 staff members. Thus, cooperative behaviour may occur if the group is of this size without punishment, reward rules or management control.

## 5. Conclusion

This study analysed a public goods game using an approach based on DQL to explore the interaction between human decision-making and the predictions of Nash equilibrium theory. Subsequently, we revealed that agents can learn to cooperate similarly to humans. Additionally, we concluded that as the number of players increase, their perceptions of the gains from cooperation and non-cooperation align. These findings have significant implications for our understanding of public goods games and human decision-making.

## Data Availability

The source code used during the current study is available in the Github repository [35] or Dryad [36]. All data was generated by this code.
